# Supplementary figures and images for: A novel CSP C-terminal epitope targeted by an antibody with protective activity against Plasmodium falciparum
Source: PLoS Pathog. 2022 Mar 28;18(3):e1010409. doi: 10.1371/journal.ppat.1010409 (PMC8989322; doi:10.1371/journal.ppat.1010409)

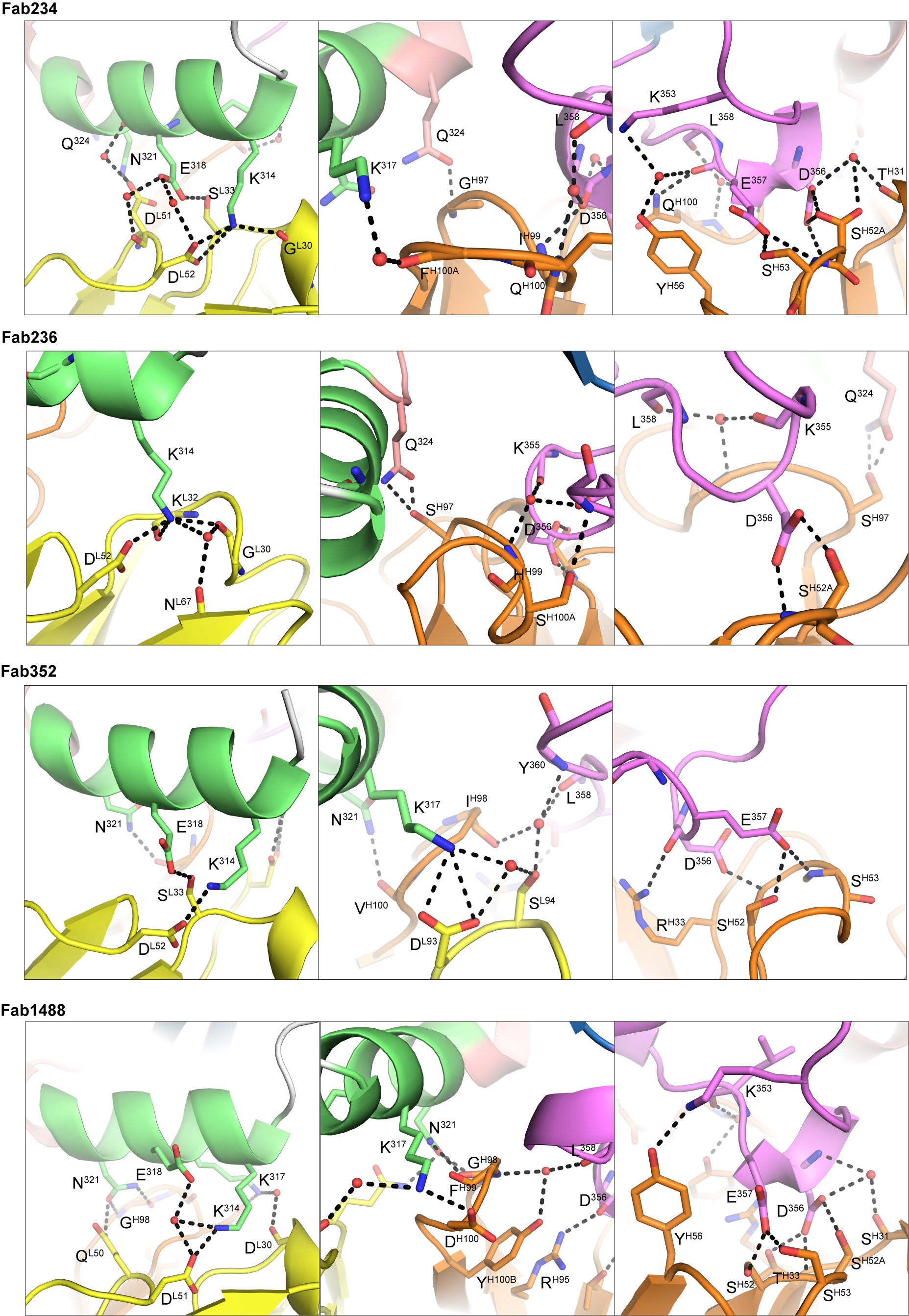

Supplement: S1 Fig — Hydrogen bonds between mAbs 234, 236, 352, 1488 and ctCSP are shown. Antibodies and ctCSP are shown in a ribbon representation, with side chains as sticks. ctCSP is colored green, salmon, and magenta for the alpha helix, CS flap, and linker region, respectively (see also Fig 2B). Antibody heavy and light chains are colored orange and yellow, respectively. Black dashes represent hydrogen bonds, whereas oxygen atoms that represent water molecules are shown as red spheres. (TIF) [file ppat.1010409.s001.tif]

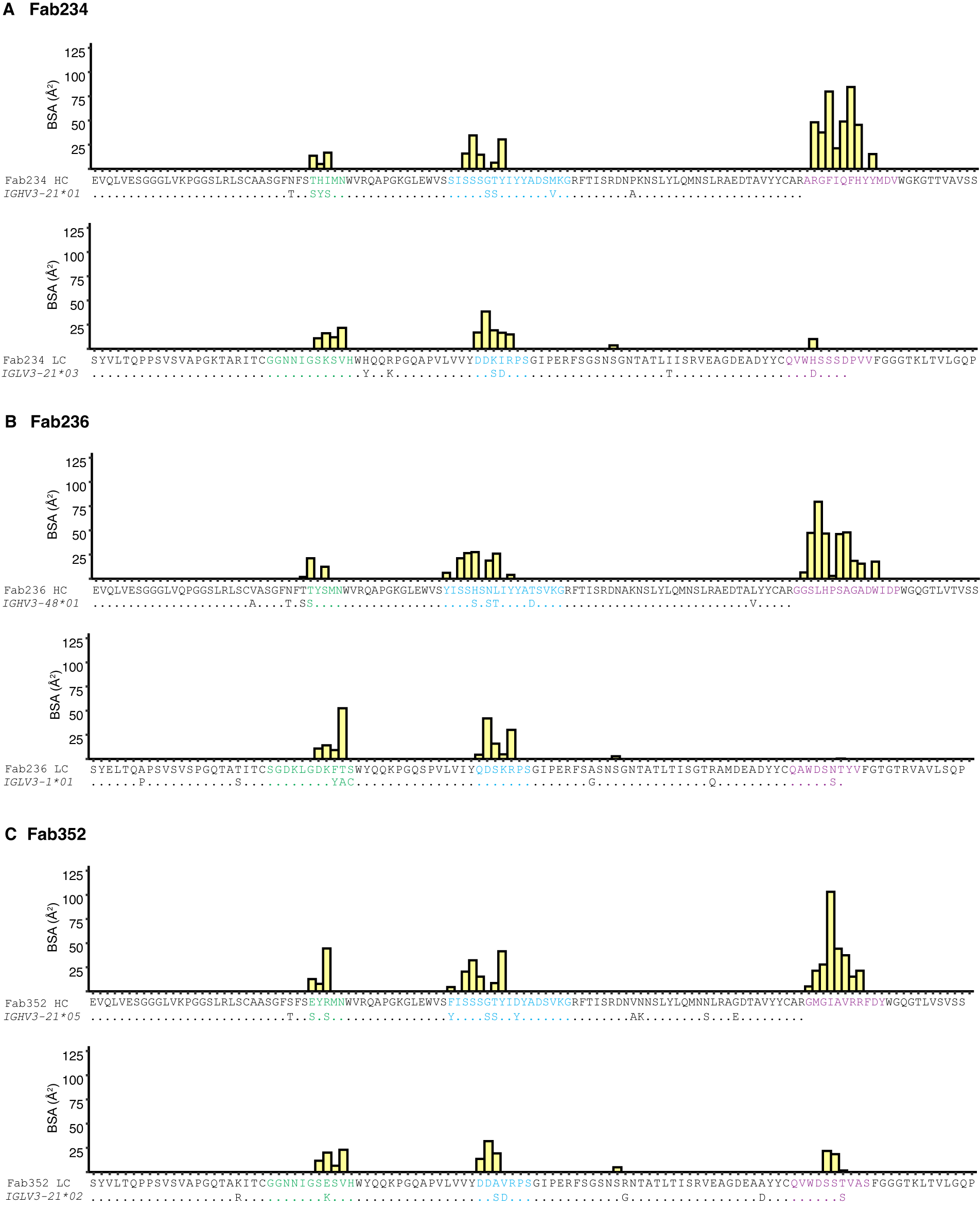

Supplement: S2 Fig — BSAs are shown in yellow bars for the heavy and light chains of (A) Fab234, (B) Fab236, and (C) Fab352. CDRs are colored in green, blue, magenta for CDR H1, H2, H3 for heavy chains or L1, L2, and L3 for light chains, respectively. Additionally, the alignment between the Fab heavy and light chain sequences and germline IGHV and IGLV gene sequences, respectively, indicates which residues are somatically mutated. (TIF) [file ppat.1010409.s002.tif]

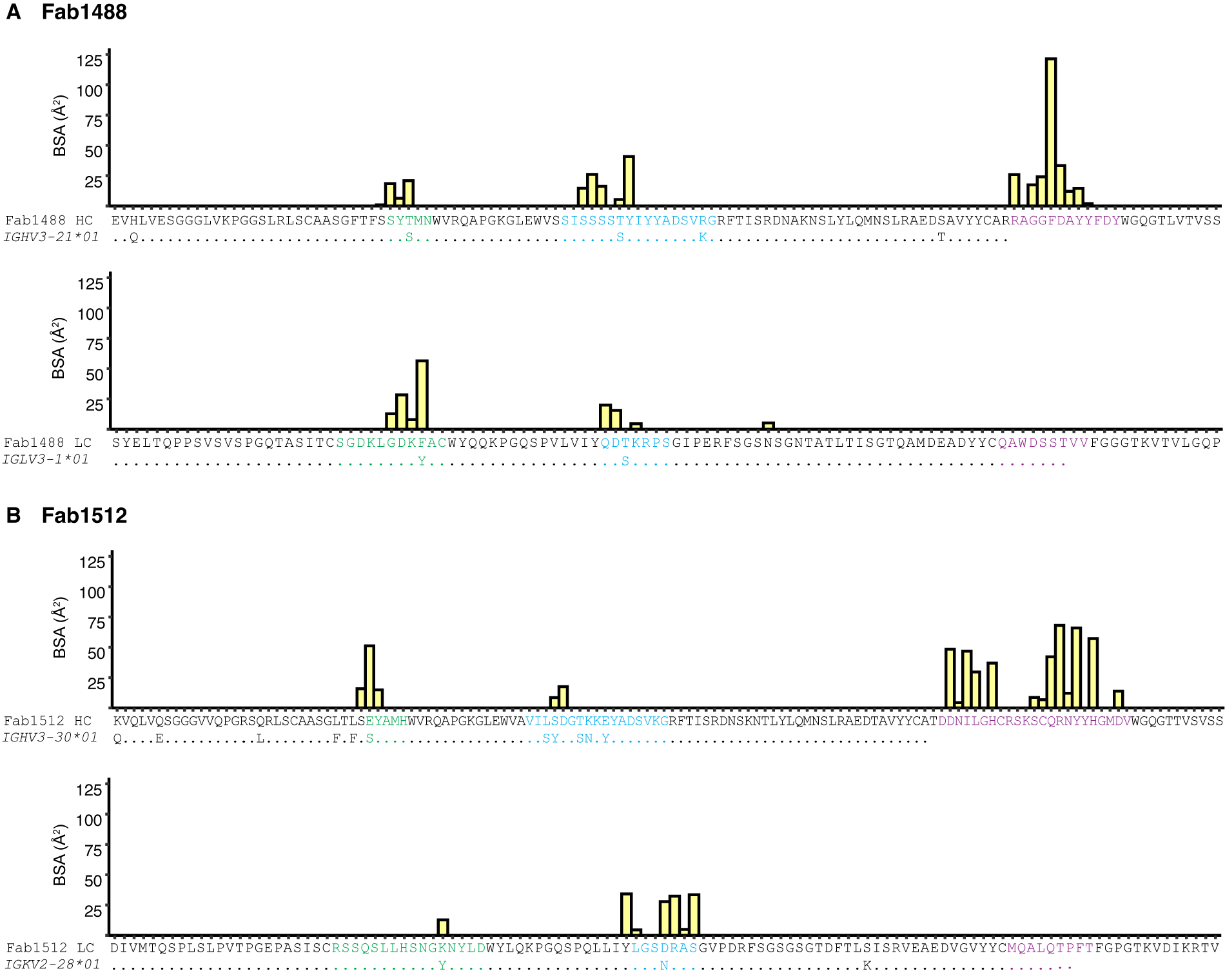

Supplement: S3 Fig — BSAs are shown in yellow bars for the heavy and light chains of (A) Fab1488 and (B) Fab1512. CDRs are colored in green, blue, magenta for CDR H1, H2, H3 for heavy chains or L1, L2, and L3 for light chains, respectively. Additionally, the alignment between the Fab heavy and light chain sequences and germline IGHV and IGLV or IGKV gene sequences, respectively, indicates which residues are somatically mutated. (TIF) [file ppat.1010409.s003.tif]

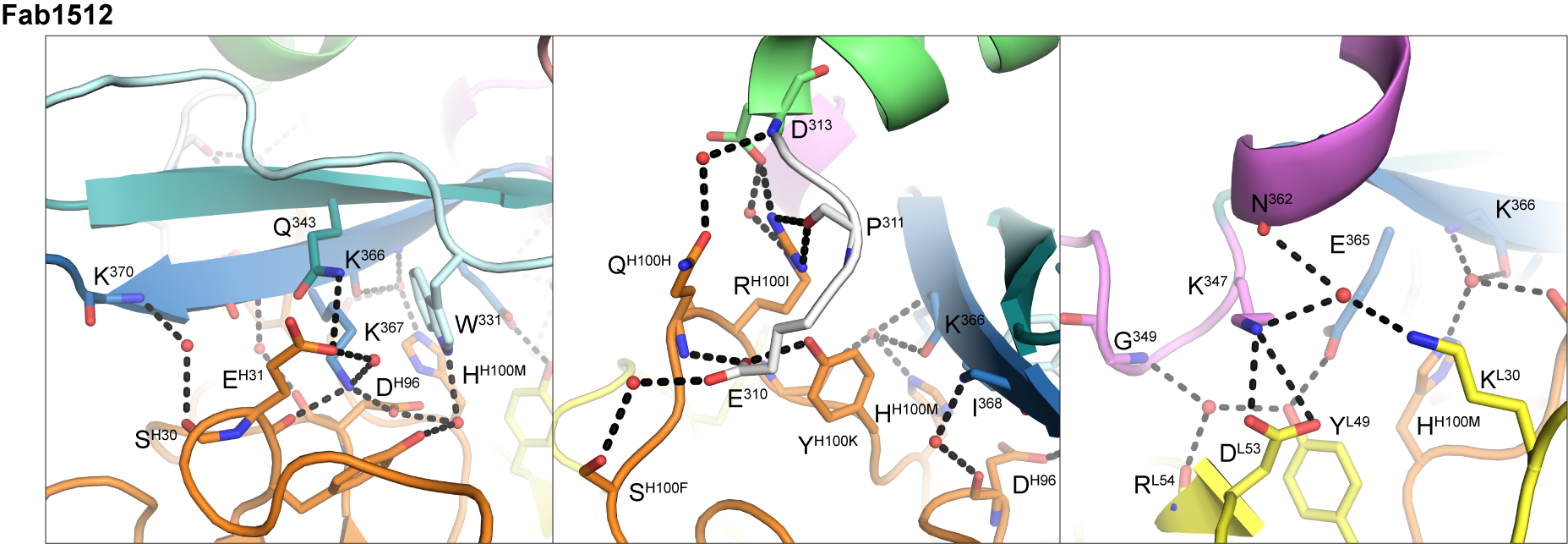

Supplement: S4 Fig — Hydrogen bonds between mAb1512 and ctCSP are shown. The antibody and ctCSP are shown in ribbon representation, with side chains as sticks. ctCSP is colored green and magenta for the alpha helix and CS flap regions, and different shades of blue for the three TSR homology region strands (see also Fig 2B). Antibody heavy and light chains are colored orange and yellow, respectively. Black dashes represent hydrogen bonds, whereas oxygen atoms that represent water molecules are shown as red spheres. (TIF) [file ppat.1010409.s004.tif]

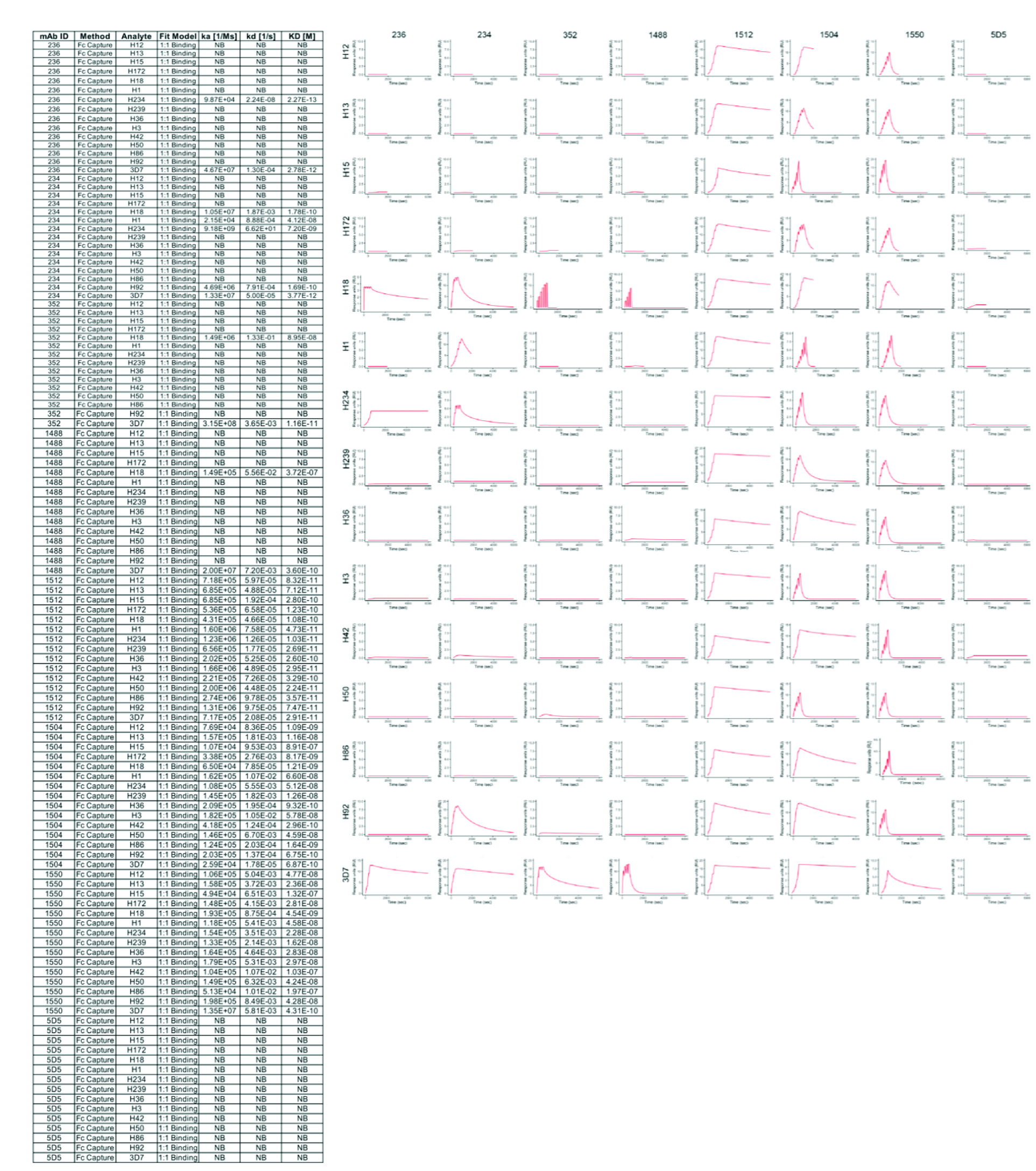

Supplement: S5 Fig — Panel of the binding of ctCSP peptides to mAbs via a Fc-capture, single cycle, multi-injection method. Association and dissociation constants were calculated through a 1:1 Langmuir binding model using BIAevaluation software. NB = No binding. SPR sensorgrams for ctCSP peptide binding displaying best global fits. Antibodies were captured on anti-human IgG (Fc) antibody immobilized on a CM5 chip and varying concentrations of ctCSP were injected using a single cycle method. Sensorgrams in resonance units (RUs) plotted against time are shown. (TIF) [file ppat.1010409.s005.tif]

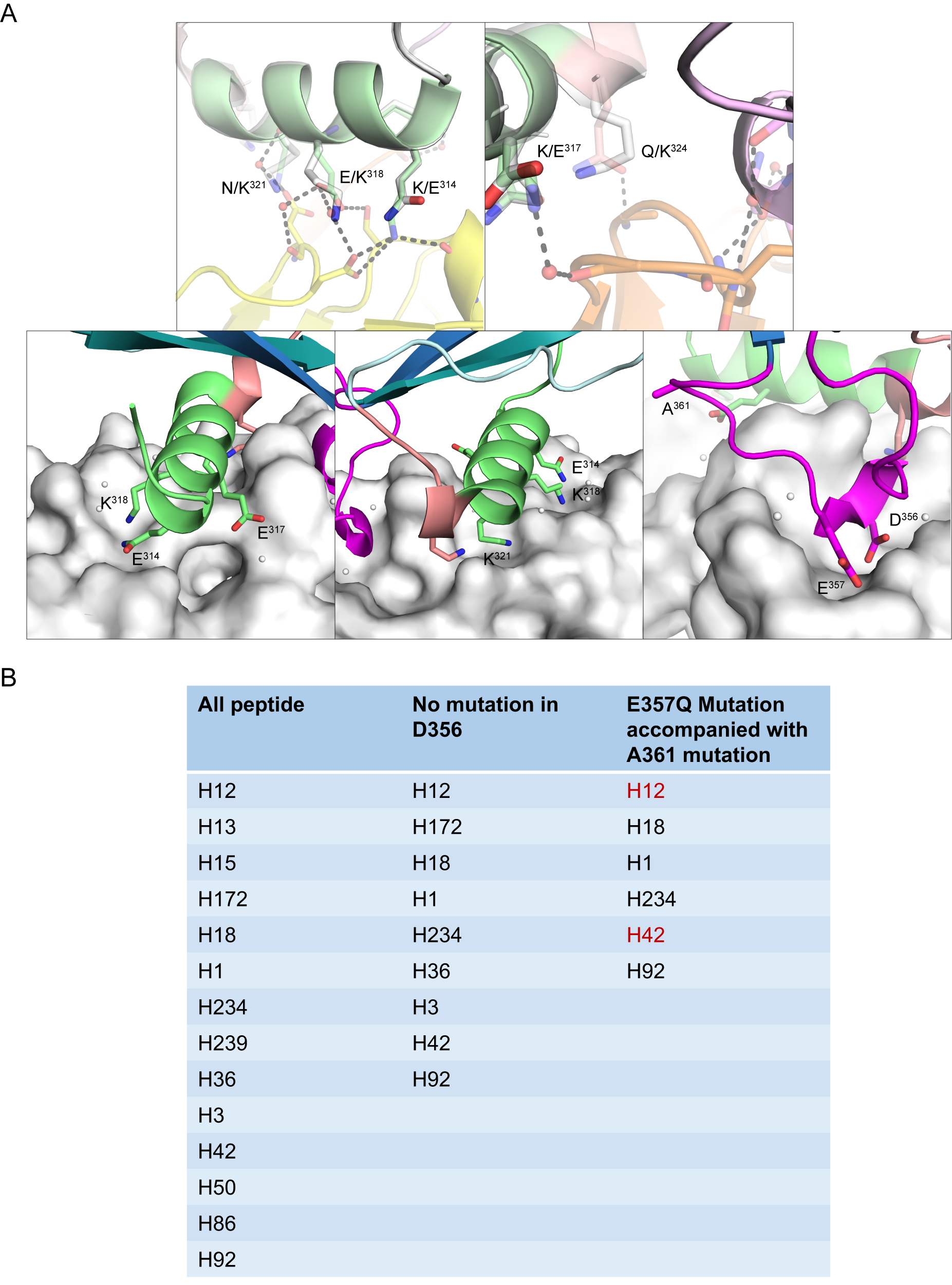

Supplement: S6 Fig — (A) The side chains of residues in the crystal structure of Fab234 in complex with the 3D7 peptide were manually mutated to the side chains of H234 in PyMOL. Top panel: the crystal structure of Fab234 in complex with 3D7 ctCSP (colored and represented as in S1 Fig) is overlaid with the computationally mutated H234 ctCSP (grey ribbons with side chains as sticks). The residues are labeled in the format of A/B where A is an H234 residue and B is a 3D7 residue. Bottom panel: Fab234 (white surfaces) in complex with the computationally mutated H234 ctCSP (colored and represented as in S1 Fig). (B) Table showing successive filtering of all peptides based on two criteria. The first column lists all peptides. The second column lists all peptides that has no mutation in residue D356, and the last column lists all peptides that has E357Q mutation with a mutation in residue A361. All peptides in last column, except for, H12 and H92 (in red), binds to mAb234 (see also Fig 3). (TIF) [file ppat.1010409.s006.tif]

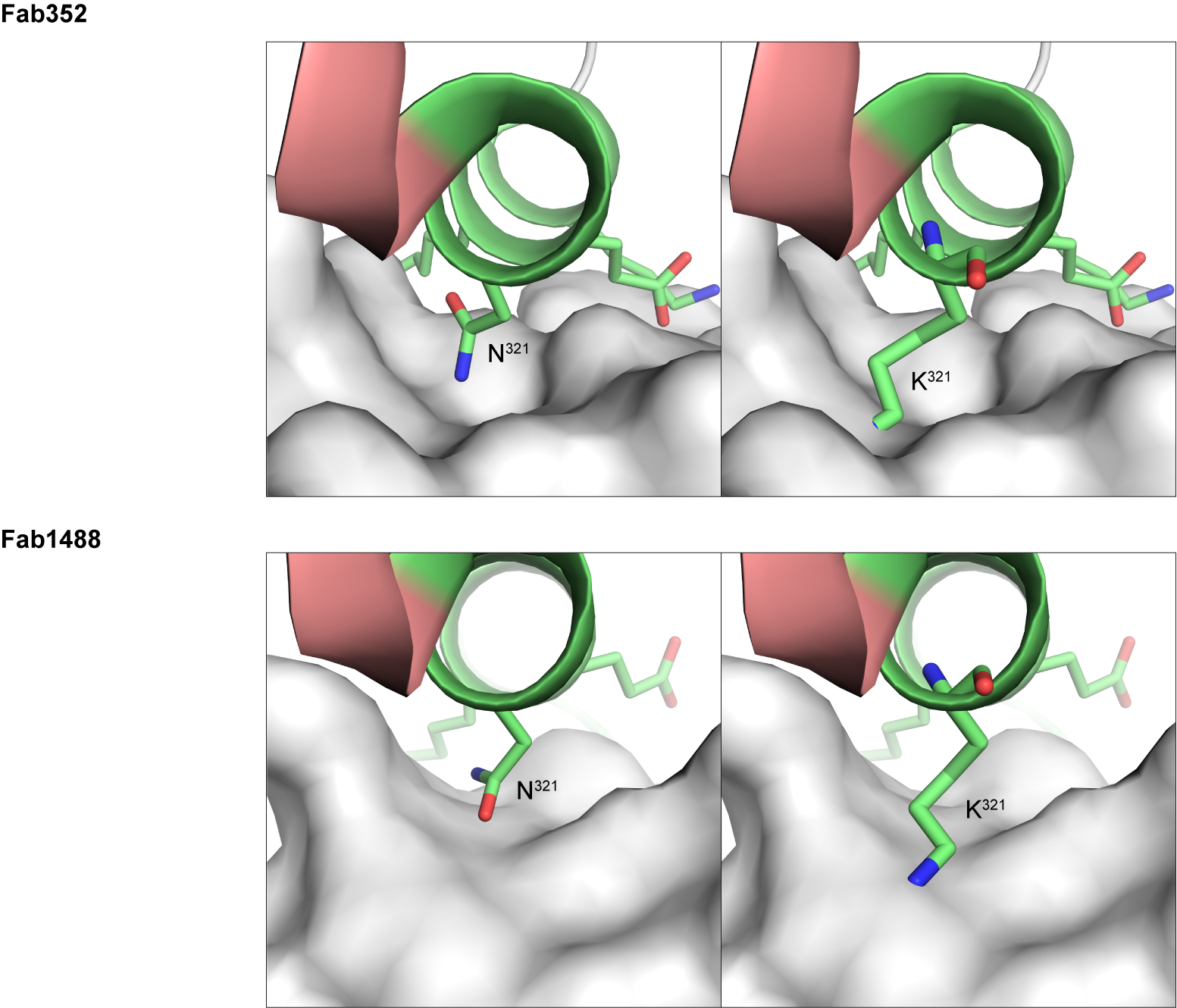

Supplement: S7 Fig — The side chain of N321 in the crystal structure each Fab in complex with the 3D7 peptide was manually mutated in PyMOL to K321, which is found in some haplotypes, The antibody is shown as white surfaces, whereas the Th2R alpha-helix is shown in ribbons with side chains as sticks. (TIF) [file ppat.1010409.s007.tif]

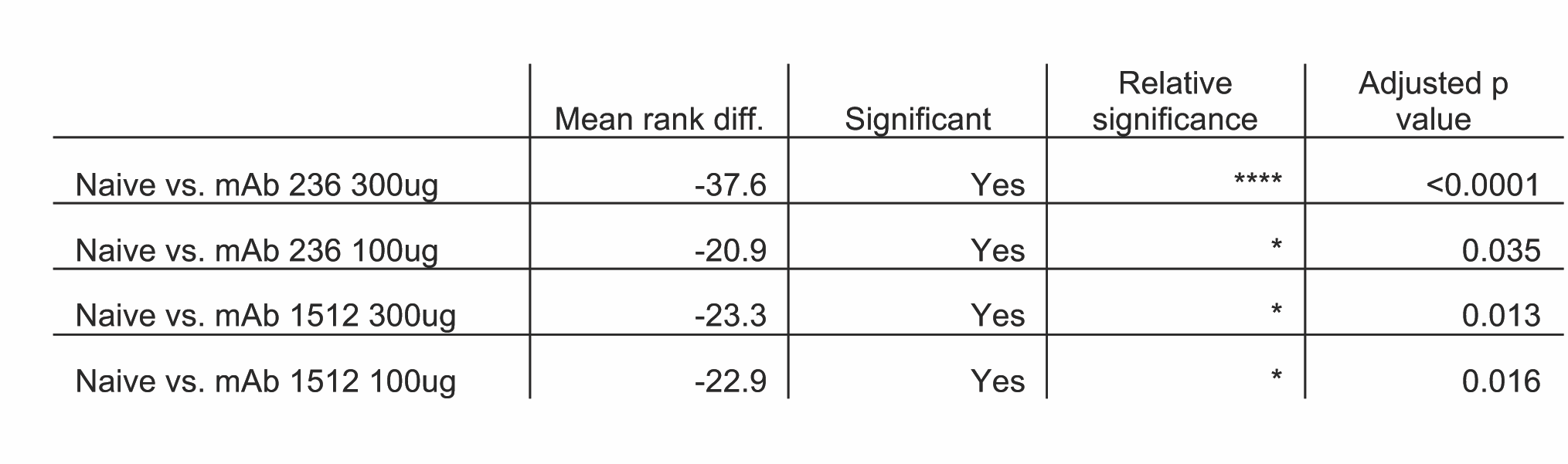

Supplement: S8 Fig — Mice, five per group in three separate experiments, were examined for % inhibition, which is the percentage of parasite burden in the liver in each mouse as compared to the mean of the untreated control group (100%). The bars in Fig 5 represent geometric mean. mAb1512 at both 300 μg and 100 μg and mAb236 at 300 μg and 100 μg exhibited statistically significant inhibition compared to the control group (p < 0.035 by Kruskal-Wallis test). Mean rank difference represents the difference in the geometric mean between naive and mAb. (TIF) [file ppat.1010409.s008.tif]

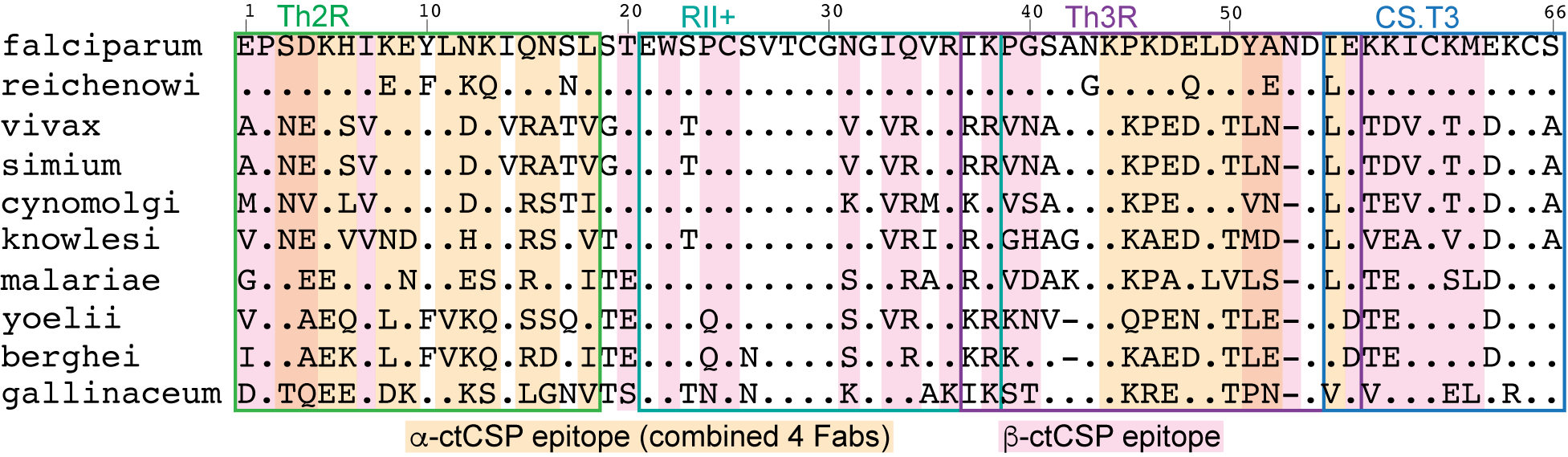

Supplement: S9 Fig — The alignment of ctCSP PfCSP sequences from different species was adapted from Doud et al. [15]. The Th2R, RII+, Th3R, and CS.T3 regions are enclosed in green, cyan, magenta, and blue boxes respectively. The combined α-ctCSP epitopes from mAbs 234, 236, 352, and 1488 are highlighted in yellow, whereas the β-ctCSP epitope of mAb 1512 is shown in pink. Human Plasmodium parasites: P. falciparum, P. vivax, P. malariae, and P. knowlesi (also infects non-human primates); non-human primate Plasmodium parasites: P. reichenowi, P. simium, and P. cynomolgi; avian Plasmodium parasite: P. gallinaceum; mouse Plasmodium parasites: P. yoelii and P. berghei. (TIF) [file ppat.1010409.s009.tif]
