# Supplementary material for: A novel CSP C-terminal epitope targeted by an antibody with protective activity against Plasmodium falciparum
Source: PLoS Pathog. 2022 Mar 28;18(3):e1010409. doi: 10.1371/journal.ppat.1010409 (PMC8989322; doi:10.1371/journal.ppat.1010409)
Supplement: S1 Table — (DOCX) [file ppat.1010409.s010.docx]

**S1 Table. X-ray data collection and refinement statistics.**

| **Data collection** | **Fab234-ctCSP** | **Fab236-ctCSP** | **Fab352-ctCSP** | **Fab1488-ctCSP** | **Fab1512-ctCSP** |
| --- | --- | --- | --- | --- | --- |
| Beamline | SSRL12-2 | APS23-IDB | APS23-IDB | APS23-IDB | APS23-IDB |
| Wavelength (Å) | 0.97946 | 1.03320 | 1.03320 | 1.03320 | 1.03324 |
| Space group | P4_1_2_1_2 | C2 | P2_1_ | P1 | P2_1_2_1_2_1_ |
| Unit cell parameters (Å, °) | a=b=115.69, c=119.30 | a=130.14, b=45.56, c=201.09 | a=84.06, b=70.50, c=105.96 | a=40.69, b=69.34, c=93.21 | a=73.01, b=84.15, c=90.22 |
|  | α=β=γ=90 | α=γ=90, β=94.0 | α=γ=90, β=100.2 | α=85.1, β=78.1, γ=79.2 | α=β=γ=90 |
| Resolution (Å) | 50.00-2.15 (2.19-2.15)^a^ | 50.00-2.40 (2.44-2.40) ^a^ | 50.00-2.80 (2.85-2.80) ^a^ | 50.00-1.82 (1.85-1.82) ^a^ | 50.00-1.76 (1.79-1.76) ^a^ |
| Unique Reflections | 43,634 (2,124) ^a^ | 46,380 (1678) ^a^ | 30,400 (1,420) | 82,577 (3,628) | 55,594 (2,685) |
| Redundancy | 5.5 (4.6) ^a^ | 5.2 (3.1) | 2.9 (2.6) | 2.9 (1.5) | 11.5 (6.9) |
| Completeness (%) | 97.9 (97.2)a | 96.7 (71.1) | 98.5 (91.4) | 95.3 (84.0) | 99.8 (97.4) |
| <I/σI> | 21.2 (1.8) ^a^ | 13.7 (1.6) | 5.6 (1.1) | 10.6 (1.0) | 24.0 (1.8) |
| Rsym^b^ (%) | 9.2 (94.0) ^a^ | 12.0 (46.3) | 20.7 (86.0) | 9.5 (61.2) | 10.8 (90.1) |
| Rpim^b^ (%) | 4.2 (45.8) ^a^ | 5.5 (25.4) | 14.1 (61.9) | 6.5 (61.2) | 3.3 (35.7) |
| CC1/2^c^ (%) | 92.8 (73.0)^a^ | 94.6 (82.8) | 84.9 (55.3) | 89.1 (63.0) | 96.0 (76.9) |
| **Refinement statistics** |  |  |  |  |  |
| Resolution (Å) | 39.08-2.15 | 42.24-2.40 | 41.36-2.80 | 39.21-1.82 | 31.40-1.76 |
| Reflections (work) | 43,550 | 46,326 | 30,292 | 82,511 | 55,495 |
| Reflections (test) | 2,243 | 2,436 | 1,495 | 3,910 | 2,769 |
| Rcryst^d^ / Rfree^e^ (%) | 21.8/26.5 | 21.8/24.9 | 22.5/27.6 | 20.7/25.5 | 17.7/20.5 |
| No. of atoms |  |  |  |  |  |
| Fab | 6,227 | 12,397 | 12,455 | 12,429 | 6,891 |
| ct-CSP | 993 | 1,781 | 1,843 | 1,958 | 1,004 |
| Water | 193 | 139 | 49 | 307 | 528 |
| Average B-value (Å^2^) |  |  |  |  |  |
| Fab | 46 | 45 | 43 | 38 | 24 |
| ct-CSP | 61 | 61 | 61 | 44 | 26 |
| Waters | 40 | 36 | 30 | 37 | 30 |
| Wilson B-value | 37 | 40 | 44 | 25 | 20 |
| RMSD from ideal geometry |  |  |  |  |  |
| Bond length (Å) | 0.013 | 0.009 | 0.004 | 0.008 | 0.010 |
| Bond angle (°) | 1.07 | 0.96 | 0.69 | 1.14 | 1.04 |
| Ramachandran statistics^f^ |  |  |  |  |  |
| Favored (%) | 98.13 | 96.48 | 94.32 | 97.23 | 98.44 |
| Outliers (%) | 0.21 | 0.10 | 0.62 | 0.21 | 0.00 |
|  |  |  |  |  |  |

^a^ Numbers in parentheses refer to the highest resolution shell.

^b^ *R*_sym_ = Σ*_hkl_* Σ*_i_* | I*_hkl,i_* - <I*_hkl_*> | / Σ*_hkl_* Σ*_i_* I*_hkl,i_* and R*_pim_* = Σ*_hkl_* (1/(n-1))^1/2^ Σ*_i_* | I*_hkl,i_* - <I*_hkl_*> | / Σ*_hkl_* Σ*_i_* I*_hkl,i_*, where I*_hkl,i_* is the scaled intensity of the i^th^ measurement of reflection h, k, l, <I*_hkl_*> is the average intensity for that reflection, and *n* is the redundancy.

^c^ CC_1/2_ = Pearson correlation coefficient between two random half datasets.

*^d^ R*_cryst_ = Σ*_hkl_* | *F*_o_ - *F*_c_ | / Σ*_hkl_* | *F*_o_ | x 100, where *F*_o_ and *F*_c_ are the observed and calculated structure factors, respectively.

^e^ *R*_free_ was calculated as for *R*_cryst_, but on a test set comprising 5% of the data excluded from refinement.

^f^ From MolProbity [1]*.*

**Reference**

1. Chen VB, Arendall WB, 3rd, Headd JJ, Keedy DA, Immormino RM, Kapral GJ, et al. MolProbity: all-atom structure validation for macromolecular crystallography. Acta Crystallogr D Biol Crystallogr. 2010;66(Pt 1):12-21.
