# Supplementary material for: A novel CSP C-terminal epitope targeted by an antibody with protective activity against Plasmodium falciparum
Source: PLoS Pathog. 2022 Mar 28;18(3):e1010409. doi: 10.1371/journal.ppat.1010409 (PMC8989322; doi:10.1371/journal.ppat.1010409)
Supplement: S2 Table — (DOCX) [file ppat.1010409.s011.docx]

**S2 Table. Buried surface area (BSA) and CDRH3 length of mAbs analyzed in this study.**

| **mAb** | **HC BSA (Å^2^)** | **LC BSA (Å^2^)** | **Total BSA (Å^2^)** | **CDRH3 length** |
| --- | --- | --- | --- | --- |
| 234 | 519 | 180 | 699 | 13 |
| 236 | 494 | 187 | 681 | 15 |
| 352 | 463 | 174 | 637 | 11 |
| 1488 | 401 | 151 | 552 | 12 |
| 1512 | 548 | 150 | 698 | 23 |
| 1710 [1] | 482 | 230 | 712 | 14 |

**Reference**

1. Scally SW, Murugan R, Bosch A, Triller G, Costa G, Mordmüller B, et al. Rare PfCSP C-terminal antibodies induced by live sporozoite vaccination are ineffective against malaria infection. J Exp Med. 2017;215(1):63-75.
