# Supplementary material for: A novel CSP C-terminal epitope targeted by an antibody with protective activity against Plasmodium falciparum
Source: PLoS Pathog. 2022 Mar 28;18(3):e1010409. doi: 10.1371/journal.ppat.1010409 (PMC8989322; doi:10.1371/journal.ppat.1010409)
Supplement: Attachment — Submitted filename: Beutler_Pholcharee_response-to-reviewer-comments_v4.docx [file ppat.1010409.s012.docx]

Editor Comments

1. Please describe the magnitude of the polyclonal response to ctCSP compared to NANP repeat in the samples studied and relative frequency of anti ctCSP mAbs compared to anti NANP mAbs.

Please see response to Part II reviewer #1 point 1 below.

2. The authors speculate that conformational changes induced by α-ctCSP mAb binding may hinder β-ctCSP mAb recognition. This does not appear to be currently supported by the structural studies in the manuscript. Please provide either more discussion highlighting that the claim is not based on structural data or additional structural information to support this claim?

Please see response to Part II reviewer #1 point 4 below.

3. Is blood stage parasitemia prevented in any of the mice treated with mAbs 236 and 1512? If unlikely, can the authors please make this clear in the discussion in comparisons to expectations from previous results.

We have included discussion concerning parasitemia of mAbs 236 and 1512 on page 15 lines 367-370.

*“It has been shown that the breadth of ctCSP antibody response following RTS,S/AS01_B_ immunization is strongly associated with protection as assessed by parasitemia (27). Therefore, it is not unreasonable to hypothesize that the ctCSP mAbs here would also reduce parasitemia in the utilized model.”*

Reviewer's Responses to Questions

**Part II – Major Issues: Key Experiments Required for Acceptance**
Please use this section to detail the key new experiments or modifications of existing experiments that should be absolutely required to validate study conclusions.

Generally, there should be no more than 3 such required experiments or major modifications for a "Major Revision" recommendation. If more than 3 experiments are necessary to validate the study conclusions, then you are encouraged to recommend "Reject".

**Reviewer #1**: Major points:

1. It would be important to state the way the ctCPS mAbs were isolated (from MBCs or PBs or PCs), the number of mAbs that were screened, and the down-selection process, if any. If possible, it would also be informative to understand the magnitude of the polyclonal response to ctCSP compared to the NANP repeat in the samples studied.

We have now added further details on antibody isolation and selection in the text on page 7, lines 157-173.

*“All vaccinated subjects in this trial showed positive titers for PfCSP, NANP_6_, and C-terminal domain (ctCSP) reactive antibodies in sera following vaccination. Sequencing of the variable regions of paired heavy and light chain messenger RNA was conducted from peripheral blood mononuclear cell (PBMC)–sorted plasmablasts sampled 7 days after the third immunization (37). Results of the somatic hypermutation frequency have previously been reported (35) and a full analysis of expressed antibody sequences is being prepared for publication. Sequences for expression of mAbs were selected to sample a variety of IgG lineages from vaccinee plasmablast responses based on multiple properties, including degree of cellular and/or clonal expansion, level of somatic hypermutation, and sequence convergence among vaccinees. For this study, we did not attempt to evaluate if these antibodies were necessarily characteristic of the level or specificity of the antbodies present in the sera of individual or grouped subjects. Expressed mAbs were screened by ELISA for reactivity to full-length CSP, followed by NANP_6_, Pf16 C-term peptide, and HBsAg as previously described (35). From 369 antibody lineages screened, 20 bound to ctCSP. We structurally and functionally characterized some of the other antibodies to the NANP repeats from this vaccine trial in previous studies (23, 24, 32, 33). The sequences studied here are specifically reactive to Pf16 C-term peptide in ELISA and further selected to represent families with different VH and VL gene usage.”*

2. The confocal microscopy section of the study should be improved. For example, Fig 4 only shows staining data for a single parasite with each mAb. The data as presented does not allow to determine whether this staining pattern is the only one observed, or whether there was heterogeneity in the reactivity. If indeed heterogeneity was observed, it would be valuable to know whether the pattern described represents the majority or minority of PfCSP reactogenicity.

The heterogeneity of the imaged sporozoites has been addressed on page 12, lines 302-303.

*“These images are representative of a majority of all stained sporozoites. Of all organisms, approximately 30% remained unstained.”*

3. In addition, based on the methods provided, it appears that the sporozoites characterized are fixed and permeabilized - 4% PFA is standard fixative. Strong DAPI staining in Fig 4 is another strong indication that the cell membrane is no longer intact and that the sporozoites imaged are likely dead. As such, the mention of “live sporozoites” in lines 258-260 is inadequate, and should be described as “fixed sporozoites”. If indeed the sporozoites analyzed and imaged are believed to be “live”, it will be important to provide additional data to support such a claim.

Many thanks for the comment. The sporozoites used in the experiment in Fig 4 were indeed ‘fixed’ sporozoites. We have replaced the term ‘live’ with ‘fixed’ sporozoites.

4. Through competition binding studies, the α-ctCSP-targeting mAbs presented in this study were found to inhibit β-ctCSP mAb binding, but the same cannot be said for the reverse scenario. The authors speculate that this may be due to conformational changes induced by α-ctCSP mAb binding that hinder β-ctCSP mAb recognition (lines 176-179). Can the authors comment on whether their structural studies support this hypothesis? Do the crystallized αTSR domains overlay similarly regardless of the mAb bound? Conversely, could there be a different explanation for this data? e.g. partial occlusion of epitope because of preferred orientation of antigen on biosensors, or difference in on-rate/off-rate between mAbs 1 and 2?

Speculation in the results section has been removed and discussion has been added on page 13, lines 324-328. The non-reciprocal binding suggests that there may be structural or dynamic changes, although this has not yet been validated by structural studies. Further experiments will need to be conducted to address this phenomenon.

*“The non-reciprocal competition seen in these experiments suggests binding of the 236-type mAbs may induce changes in the structure or dynamics of the ctCSP that inhibit binding of the 1512-type mAbs with the reverse not being the case, i.e. binding of 1512-type Abs does not induce changes that affect 236-type Ab binding, although structural studies to validate any changes have not yet been performed.”*

**Reviewer #2**: The study would benefit from additional clarification and/or experiments:

1. previous study by Scally at al indicated that Mabs directed against the alpha epitope region were not protective while this study show some level of inhibition with mAb236. It would be nice to repeat the in vivo protection with Mab 1710 as control to be able to compare side by side the reported findings. Additionally, adding a control anti-NANP mAb such as 317 will be of interest to compare side by side the inhibitory activity of mAbs directed against the repeat vs the C-terminus of PfCSP, even though the authors mention in the discussion that the inhibitory activity of the C-term MAb is less than that of the Mab directed against the repeat.

We feel that including another antibody at this stage would incur serious delay as it would require repeating the in vivo experiment to compare the data to mAb1710 and 317. We feel that our data are sufficient to demonstrate protective activity of mAbs to the beta epitope and, as noted by the reviewer, this activity is not as strong as the repeat region mAbs.

2. Can the authors use the structures of the mAb to the alpha epitope region to explain differences observed in binding recognition to the different peptides

Thank you for the suggestion. We have added some discussion on the differences observed in binding of α-ctCSP-specific mAbs to different haplotypes under the “β-ctCSP specific mAbs display broad binding at high affinity” (pages 11-12 lines 266-295). S6 and S7 Figs are also added to accompany the explanation provided (previous S6 and S7 Figs are now S8 and S9 Figs).

*“The structures of α-ctCSP mAbs might offer hints on some of the binding activities observed in this class of antibodies. We first noted that, besides the wild-type 3D7 peptide, mAb234 binds to four additional peptides, H18, H1, H234, and H92 (****Fig 3****). The haplotype H234 differs substantially from 3D7 at the Th2R region with six mutations, yet can still interact with mAb234 (****Fig 3****). The crystal structure suggests that the binding promiscuity of mAb234 to Th2R may be possible because the H-bonds between mAb234 and Th2R are mostly mediated by water molecules (****S1 Fig****). In fact, when we modeled replacement of the 3D7 residues in the crystal structure with H234 haplotype residues, we found that Fab234 can accommodate these mutations with no clashes and may be able to even make similar H-bonds with the mutated ct-CSP (****S6A Fig****). The surface of mAb234 may also large enough to accommodate some larger changes in a side chain, e.g., the N321K mutation (****S6A Fig****), athough that needs to be experimentally confirmed. The limiting region seems to be Th3R even though it is difficult to describe why this is the case based only on our structure of Fab234 in complex with the 3D7 peptide. Based on the binding study, however, we observed that mAb234 cannot bind to haplotypes with D356N/E mutations and does not seem to bind to haplotypes with an E357 mutation that is not accompanied by mutation at A361 (****S6B Fig****). The combination of mutations in Th2R (or overall) may also play a role as the peptides H12 and H42 do not meet the two criteria above, but still cannot interact with mAb234. Similar to mAb234, mAb236 is flexible for binding to Th2R as it also binds to the H234 haplotype (****Fig 3B****). However, binding to Th3R seems to be crucial to mAb236 since the antibody does not bind to any haplotypes that have mutations in that region (****Fig 3A****).*

*In contrast to the promiscuity in mAb234 binding to the Th2R helix, the interactions with that region seem to be critical for mAbs 352 and 1488 as these two antibodies only bind the H18 haplotype (besides 3D7), which has no mutations in Th2R except for L327I in the perimeter (****Fig 3****). One explanation for this intolerance for Th2R mutations is that mAbs 352 and 1488 have tighter binding surfaces that would clash with e.g., the N321K mutation, which is present in most haplotypes (****S7 Fig****). For haplotypes H36 and H92, which do not have N321K mutation (****Fig 3A****), we suspect that changes in residues 317 and 318 may disrupt the binding of ct-CSP to mAb352 and 1488, although it is difficult to specify how this happens without determining structures of these antibody-peptide complexes (changes in residues 317 and 318 do not seem to obviously clash with the antibody surface).”*

3. Have the authors tried to co-crystallize 1504 and 1550? Can they mention why these structures are not shown here (there are 4 for one group vs 1 for the others), especially since one 1550 has the shorter CDRH3.

This is an interesting point. We chose the antibody with the highest apparent affinity (1512) and may in the future follow up with 1504 and 1550.

Reviewer #3: No Response

**Part III – Minor Issues: Editorial and Data Presentation Modifications**
Please use this section for editorial suggestions as well as relatively minor modifications of existing data that would enhance clarity.

**Reviewer #1**: Minor points:

1. Impressively, the authors describe the crystal structures of five Fabs each in complex with the ctCSP αTSR. Does the breadth of binding of these mAbs against the panel of peptides tested by SPR correspond to the specific molecular interactions identified in the crystal structures? Providing a brief structural basis for the binding patterns across sequence diversity would further strengthen and solidify the message of this study.

Thank you for the suggestion. As also suggested by Reviewer 2, we have added some discussion on the differences observed in binding of α-ctCSP-specific mAbs to different haplotypes (pages 11-12, lines 266-296; see point 2 in response to reviewer #2 above). S6 and S7 Figs are also added to accompany the explanation provided (previous S6 and S7 Figs are now S8 and S9 Figs). This analysis further strengthens our conclusion.

2. In Fig 3a, a description of the orange-highlighted residues in the figure caption would help clarify the figure further.

The orange-highlighted residues represent residues that are the same between the α and β epitopes. A sentence was added in Fig 3a legend for clarification.

*“Residues that are present in both the α-ctCSP and β-ctCSP epitopes are highlighted in orange. The transparent surfaces overlaid with ribbons of ctCSP are also shown and the combined α-ctCSP epitopes, β-ctCSP epitope, and overlapping epitopes are colored in pink, wheat, and orange as above.”*

3. Lines 114-115: this sentence suggests that 95% protective efficacy was observed in the control group of the Phase 2 clinical study examining vaccine candidate R21.

We have corrected the error in the introduction section regarding R21 (page 5, line 111-112)

*“Efficacy levels of 74%, and 77%, in the low- and high-dose adjuvant groups, and control group respectively were observed in a 12-month follow-up period following dose 3 (11),”*

4. Lines 131 and 138: once defined, the authors should use acronyms consistently throughout the publication (i.e. “ctCSP” and “mAbs”)

Thank you. We have made corrections as suggested.

5. Line 149-150: given that the study characterizes only one β-ctCSP-specific mAb with limited protective efficacy, I suggest that the authors soften the language of this statement to avoid over-generalizing their findings.

We agree and have modified and toned down the language here (page 6, lines 146-148).

“*We further showed that one member of this new class of anti-ctCSP mAbs had anti-parasite activity by conducting an in vivo protection study in mice using transgenic P. berghei that express PfCSP, although further studies are required to generalize this finding*.”

6. Line 158: it is unclear what peptide “NANPx” is referring to.

Sorry - we were referring to the NPNA_6_ peptide and have more clearly specified this now in the text.

7. Line 214: it would be beneficial to comment on the conservation of the CS.T3 epitope in P. falciparum isolates not included in the study as well.

Sorry but the wording that we used was misleading.

*“another T-cell epitope CS.T3 (****Fig 2B****), which is highly conserved among the P. falciparum isolates examined in this study* ***(Fig 3A)****.*

It was stated later in the paper

*“we generated a panel of peptides that correspond to different ctCSP haplotypes as in the previous study (27). These peptides represent the extensive diversity of ctCSP sequences found naturally in P. falciparum strains”*

and

*“Consistent with the conserved nature of this epitope, mAb 1512 can bind with strong affinity to all 15 haplotypes of ctCSP that represent strains found across East and West Africa (30) (****Fig 3, and S5 Fig)****”*

Thus, we analyzed all of the repesentative *P. falciparum* isolate haplotypes as shown by comparing Fig. 3A and Fig. 3B and have amended the sentence on page 9, lines 224-225.

“*another T-cell epitope CS.T3 (****Fig 2B****), which is highly conserved among P. falciparum isolates (****Fig. 3A****)”*

8. Line 240: it is unclear why reference 36 is called out in this sentence.

The reference was added by mistake and has now been removed.

9. Lines 315 and 341: the authors are encouraged to streamline the Discussion section to avoid making the same statements twice. In this aspect, it would be helpful to the reader to better understand the inhibition range of the assay by mAbs if the liver burden activity of such an anti-NANP-repeat antibody could be shown directly in Fig. 5 along with the ctCSP antibodies.

We have reduced the repetition in this section and rearranged some sentences to reduce such repetition. An anti-NANP repeat was not included in these studies, although the potency of these mAbs in an identical assay was referenced on page 15, lines 361-363.

10. Lines 326-327: The claim about the low frequency of long CDRH3s needs to be supported by a reference.

Thank you - a reference has been added on page 16, line 380 (ref 48).

11. Line 373: Have the authors looked at whether all peptides are equally well folded (e.g. by circular dichroism) and therefore would be adequately recognized by antibodies that have conformational epitopes?

A good point- we have not done this but feel it is beyond the scope of this study. It will certainly be worth considering in the future.

12. Line 413: nm should be used here rather than nM.

Many thanks for catching these errors. They have now been corrected.

13. Line 430: “Advanced Photon Source”

The error has been corrected.

14. Line 755: “Immunogenetics of ctCSP-specific mAbs”

The error has been corrected.

15. Fig 1a: The first light chain V gene of mAb 352 is missing the “I” in “IGLV”. Also, what is the difference between groups labeled Fx017M and Fx017?

Many thanks for catching the error in Fig 1a. It has now been corrected. Also, Fx017 should be labeled as Fx017M. We apologize for the inconsistency, and have fixed the errors.

16. Fig 5: Assuming that the lines for each group indicate the mean, because the data are presented as % inhibition of parasite burden compared to the mean of the control group, shouldn’t the line for the control group align with the dotted line at 0%?

The values are normalized against the geometric mean of the naïve group. The mean of each group is plotted instead of geometric mean because after normalization negative values are generated and the geometric mean can no longer be used.

17. Figs S2 and S3 are missing y-axis titles, and it is also unclear why these plots have been grouped into separate figures.

Thank you. The y-axis should have been labeled as “BSA (Å^2^)”, which has now been added. Also, the plots are separated into different figures solely because of the size limit on the page. We think that it is better to separate them into two figures rather than using “S2 Fig (cont’d)”.

18. Fig S4 caption: “Hydrogen bonding networks between mAb 1512 and ctCSP”

The error was fixed as suggested.

**Reviewer #2**:

1. The authors mention that antibodies in mab 236 group could induce conformational changes, preventing binding of the beta epitope regions directed mabs but the structures don't see to show this. Can the authors add some discussion of what they think may be happening (line 176—179).

Please see our response Part II, reviewer #1 point 4.

2. The authors should refer to PfCP and not CSP.

We have changed CSP to *Pf*CSP throughout where appropriate.

3. In Fig 2A, it will be nice to color code ctCSP as in figure 2B for one of the structure.

We beg to differ here and think that the change would introduce too many colors as the antibodies are colored differently and even more colors would complicate interpretation of the figure.

4. In figure 3, it will be nice to have a surface representation showing the overlapping region and domain described in 3A (maybe 2 views with 180 deg rotation).

Thank you for a nice suggestion. We have now added two views of the surface representation of ctCSP in Figure 3.

5. Fig S2 - lacking Y axis legend

Thanks- this was also commented on by Reviewer 1. We have fixed this omission.

**Reviewer #3**:

1. Can the authors provide the relative frequency of anti ctCSP mAbs compared to anti NANP mAbs in volunteers immunized with RTS,S/AS01? Are ctCSP rare or abundant?

Please see our answer in Part II, reviewer #1 point 1 where 20 of 369 antibodies isolated were to ctCSP.

2. Line 176. The authors suggest that upon 236, 234, 352, or 1488 mAb binding to ctCSP, conformational changes may be induced to prevent binding of 1512 etc. This is potentially very interesting. Do the crystal structures support this?

We agree that would be interesting if indeed the case. As this suggestions is very speculative, we have deleted the conformational change comment here as we don’t have crystal structures to support it. Please also see the response in Part II to reviewer 1 point 4 where discussion on this point was added on page 13, lines 325-329.

3. Line 209 – The authors state that mAb1488 has a lower affinity for ctCSP compared to the other mAbs, but this hasn’t been shown yet. Perhaps reference figure 3?

We have now referenced Figure 3 in this section.

4. Line 248 – Based on the crystal structures, can the authors infer why mAb 234 showed greater breadth than the other a-ctCSP specific mAbs?

Thank you for the suggestion. As also suggested by Reviewer 2, we have added some discussion on the differences observed in binding of α-ctCSP-specific mAbs to different haplotypes under the “β-ctCSP specific mAbs display broad binding at high affinity” (pages 11-12, lines 266-296). S6 and S7 Figs are also added to accompany the explanation provided (previous S6 and S7 Figs are now S8 and S9 Figs).

5. Line 386 – Incubate not incubated

The error has been fixed.

6. Line 388 – protein was eluted “with” 200 mM

The error has been fixed.

7. In Fig 2C, some of the aa’s are mis-labeled. Please check.
8. Fab234 FH101 – FH100A and HH102 – HH100B and SL32 not labeled
9. Fab236 PH100 not labeled
10. Fab 1488 – Label residue H56
11. Fab 1710 – Label SH54? Label LCDR3 residues

Thank you very much for your careful inspection of the labels in Fig 2C. Indeed, there were some errors as identified (for points 7 to 11), and all have been corrected.

12. Figure 5 - It would be beneficial to see the data represented as total flux (photons/s) as well as inhibition, similar to Oyen D et al., 2020 Plos Path.

Figure 5 was generated directly from the primary total flux data and, therefore, provides an equivalent presentation of the data as showing total flux directly. We feel that showing the total flux in log scale will not emphasize the difference from control and would prefer to keep the current representation in Fig. 5.

13. S1 Table – need to superscript some of the a
    a. In Fab236-ctCSP column, Unique reflections (1,678)
    b. In Fab1512-ctCSP column, No. of atoms ctCSP 1,004

Thanks- these errors have been corrected as suggested.
