# Supplementary material for: A novel CSP C-terminal epitope targeted by an antibody with protective activity against Plasmodium falciparum
Source: PLoS Pathog. 2022 Mar 28;18(3):e1010409. doi: 10.1371/journal.ppat.1010409 (PMC8989322; doi:10.1371/journal.ppat.1010409)
Supplement: Attachment — Submitted filename: Beutler_Pholcharee_CtermCSP_response-to-comments_v2.docx [file ppat.1010409.s013.docx]

**Response to reviewer’s comments**

**Note to editors:** Please note that all of our funding information is included under the “Financial Disclosure” section in the manuscript. The contract numbers listed under the Acknowledgement are the contract numbers associated with the synchrotron sites at which we sent our protein crystals to. The synchrotron sites do not fund any parts of our studies.

We would like to see textual changes and addition of the raw data for total flux as requested by Reviewer 1. In particular, as there are no additional experiments required we do ask that the authors pay particular attention to the textual changes that has been requested by Reviewer 1. Below we provide a quick summary of the changes that should be addressed.

1. The authors should caveat in the Discussion that their sporozoite binding studies are performed with fixed sporozoites, and speculate on how epitope accessibility/reactivity might differ in live sporozoites, which ultimately is more relevant.

Thank you for your suggestion. We have added further discussion of the fixed sporozoite results and how they may relate to live sporozoites in the discussion on page 15, lines 398-410:

“Although these experiments utilized formaldehyde fixation, which under certain circumstances can alter the native conformation of surface proteins, it has been shown that the C-terminal domain on whole sporozoites is accessible to antibody binding in both fixed and unfixed states (37, 43). The moderate protection seen so far with ctCSP mAbs may be in part attributed to only one C-terminal antibody bound per *Pf*CSP compared to anti-NANP-repeat antibodies that are capable of binding to 10 or more sites within the NANP repeat region of *Pf*CSP (33). Our confocal microscopy data, complemented by our in-vivo findings, suggest these ctCSP epitopes are accessible on the surface of sporozoites, but whether ctCSP mAbs can achieve enhanced protection levels in combination with potent anti-NANP/junction mAbs in humans has yet to be explored.”

2. If you are unable to show that the crystallised TSR domains are conformationally different upon mAb binding, please remove the hypothesis around conformational change in ctCSP induced by α-ctCSP-targeting mAbs as requested.

We previously addressed a previous version of this comment and indeed revised our paper to tone down the conformational change hypothesis and also present alternative possibilities (dynamics) for the observation of one antibody blocking another even though their binding sites do not overlap. We alluded to this hypothesis as part of the discussion on page 13, lines 341-345 in the previous version.

“The non-reciprocal competition seen in these experiments suggests binding of the 236-type mAbs may induce changes in the structure or dynamics of the ctCSP that inhibit binding of the 1512-type mAbs with the reverse not being the case, i.e. binding of 1512-type Abs does not induce changes that affect 236-type Ab binding although structural studies to validate any changes have not yet been performed”

We have now reworded that part of the discussion to remove any mention of conformational change or other hypotheses to explain the experimental observations on page 13, 344-345.

“The non-reciprocal competition seen in these experiments suggests that binding of the 236-type mAbs to ctCSP inhibits binding of the 1512-type mAbs with the reverse not being the case, i.e. binding of 1512-type Abs does not affect 236-type Ab binding. Studies are underway to determine the basis of this non-reciprocal binding between these two groups of anti-ctCSP antibodies.”

3. Please provide the raw data for the total flux (photons) as you have previously done for Oyen et al 2020.

We have provided the plot of the total flux as an additional panel (B) in Figure 5. The figure legend was also amended to reflect this change.
